# A Compound Fault Diagnosis for Rolling Bearings Method Based on Blind Source Separation and Ensemble Empirical Mode Decomposition

**DOI:** 10.1371/journal.pone.0109166

**Published:** 2014-10-07

**Authors:** Huaqing Wang, Ruitong Li, Gang Tang, Hongfang Yuan, Qingliang Zhao, Xi Cao

**Affiliations:** 1 School of Mechanical and Electrical Engineering, Beijing University of Chemical Technology, Beijing, China; 2 School of Information Science and Technology, Beijing University of Chemical Technology, Beijing, China; College of Mechatronics and Automation, National University of Defense Technology, China

## Abstract

A Compound fault signal usually contains multiple characteristic signals and strong confusion noise, which makes it difficult to separate week fault signals from them through conventional ways, such as FFT-based envelope detection, wavelet transform or empirical mode decomposition individually. In order to improve the compound faults diagnose of rolling bearings via signals’ separation, the present paper proposes a new method to identify compound faults from measured mixed-signals, which is based on ensemble empirical mode decomposition (EEMD) method and independent component analysis (ICA) technique. With the approach, a vibration signal is firstly decomposed into intrinsic mode functions (IMF) by EEMD method to obtain multichannel signals. Then, according to a cross correlation criterion, the corresponding IMF is selected as the input matrix of ICA. Finally, the compound faults can be separated effectively by executing ICA method, which makes the fault features more easily extracted and more clearly identified. Experimental results validate the effectiveness of the proposed method in compound fault separating, which works not only for the outer race defect, but also for the rollers defect and the unbalance fault of the experimental system.

## Introduction

A rolling bearing is one of the most widely used components in rotating machinery, whose running state directly affects the accuracy, reliability and service life of the whole machine. Therefore, the condition monitoring and fault diagnosis of a rolling bearing has extremely vital significance, and it is also very important to guarantee the production efficiency and the plant safety in modern enterprises [1].

Vibration signal detection is generally an effective method for fault diagnosis of rolling bearings. Ideally, it is better if a vibration signal contains only one defect when it is measured by an acceleration sensor under low-noise condition. In this case, features of the bearing defect can be extracted by Fast Fourier Transformation (FFT) comparing with the characteristic frequencies of the bearing. This approach can mainly be applied when the fault feature is relatively obvious. However, in practice, most bearing faults are often compounded by the outer-race defect, the inner-race defect or the rollers defect. Especially, in some cases, some strong noises may be mixed into a fault signal, which may lead to misrecognition of the useful information for equipment condition monitoring and fault diagnosis.

In order to solve the problem issued above and improve the identification of fault types and the monitoring of rotating machinery’s running state, it is critically important to separate the compound faults from measured signals. Blind Source Separation (BSS) developed by Herault [2] provides a new way to help solving the problem. BSS is a kind of new technique aiming at extraction of individual signal from mixed ones. In recent years, BSS problem becomes a popular issue in the field of unsupervised neural learning and statistical signal processing, especially on the theory itself and its further applications in practice. For example, Canonical Correlation Analysis (CCA) is applied to reveal underlying components with maximum autocorrelation from fMRI data [3]–[4]. Developed with BSS, without requirements of prior information about mixed signals under its original statistically independent sources, a so-called independent component analysis (ICA) has become a powerful solution to the problem of blind source separation. With the approach, several assumptions have been set up to effectively separate independent source signals: (1) source signals are statistically independent; (2) the number of sensors is greater than or equal to that of source signals. If the number of sensors is less than that of source signals, it is commonly called Overcomplete ICA Algorithm; (3) source signals meet non-Gaussian distribution. To solve this problem, Lewicki *et.al.* [5]–[7] proposed a statistical model based on the shortest path and nature gradient. Subsequently, Waheed *et.al.* [8]–[10] put forward an algebraic overcomplete independent component analysis (AICA) and geometric overcomplete independent component analysis (Geo-ICA). Up to now, ICA has attracted considerable attention for its potential applications in a variety of research fields, such as biomedical signal processing [11]–[13], image processing and recognition [14]–[17], financial data analysis and prediction [18]–[19], speech separation [20] and face recognition [21] and so on.

In the field of equipment condition monitoring and fault diagnosis, ICA is a kind of new approach to separate vibration signals and extract fault features measured by acceleration sensors. For example, to identity different types of faults accurately and rapidly, based on kernel independent component analysis (KICA) and sparse support vector machine (SVM), Ma *et.al.* [22] proposed new approaches for complex industrial process monitoring and fault diagnosis. Atmaja *et.al.* [23] combined ICA with instantaneous frequency (IF) to detect simultaneous machinery faults using sound mixture emitted by machines. Arifianto [24] evaluated the independent component analysis techniques for remote condition monitoring by analyzing sound emitted from the machines.

In practice, a fault signal in operating equipment mostly appears as a shock sequence “rhythm”, such as inner-race defect, outer-race defect and rollers defect of rolling bearings, *etc*. Hence the vast majority of these signals obey non-Gaussian distribution. In addition, the generation of vibratory sources in rotation machines is independent, for example, causes leading to outer-race defect and rollers defect are independent. Therefore, vibration signals are usually regarded as being statistically independent [25]. In order to fulfill the ICA requirement that the number of sensors is greater than or equal to that of source signals, the number of sensors should be increased as far as possible to collect multichannel signals at a same time. However, various factors, such as unknown source signals, complexity of the transmission channel, restriction of sensor installation location and experimental cost problem *etc.*, have brought certain difficulties to the equipment condition monitoring and fault diagnosis.

Aiming at solving this problem, a single channel signal can be decomposed to multichannel signals by new tools, such as wavelet transform (WT), local mean decomposition (LMD), and empirical mode decomposition (EMD). EMD is an adaptive and efficient method proposed by Huang *et.al.* [26], which is to decompose nonlinear and non-stationary signals into intrinsic mode functions (IMF) that can be used as input matrix of ICA. Recently, some researchers have made much more beneficial attempts combined EMD with ICA. For example, to construct virtual noise channels used as input matrix of ICA, EMD was used to decompose a single vibration signal to IMF restructured based on mutual cross correlation criterion [27]–[31]. And some researches have also been done to extract fault feature of rolling bearing combing wavelet transform with ICA. For example, Senguler *et.al.* [32] applied ICA based on wavelet package analysis to extract informative trend for determining the development of bearing damage. Qiao *et.al.* [33]–[34] used discrete wavelet transform method combined with ICA theory to separate fault signal from background noise signal.

In summary, it is feasible to denoise and extract bearing fault features from weak signals by ICA technique. However, the methods mentioned above are mainly based on single fault signals. Actually, vibration signals collected by an acceleration sensor generally contain a variety of fault signals when a rotating mechanical failure occurs. Therefore, in fault diagnosis and condition monitoring for rotating machinery, it is extremely significant to employ fewer sensors to separate the single fault signal from mixed signals.

In order to diagnose faults effectively, and separate the compound faults for rotating machinery in steady operating conditions, this paper proposes a novel feature extraction method from vibration signals for rolling bearing based on blind source separation theory. First, a single channel vibration signal is decomposed into IMF by EEMD to obtain multichannel signals. Second, select IMF based on a cross correlation criterion. Third, the envelop signals of the selected IMF are used as the input matrix ICA. Finally, the compound faults can be separated, and its features can be identified. In addition, comparisons are also made among the conventional FFT-based envelope detection, the wavelet analysis, the EEMD, and the proposed method to verify the effectiveness.

## Basic Theory

### 2.1 EEMD theory

EMD [35]–[36] is very suitable for decomposing nonlinear and nonstationary time series, which can adaptively represent the local characteristics of a given signal. The main idea of EMD is to decompose a time series data into a sum of oscillatory functions, namely, intrinsic mode functions (IMF). IMF should satisfy the following two conditions: (1) in the whole time series, the difference between numbers of the extrema and the zero-crossing must be equal to zero or one; (2) at any point, the mean value of the upper and lower envelopes is zero. The process for obtaining the IMF decomposition is known as “sifting,” with the following steps.

#### Step 1

Identify all the local extrema including the minimum values and maximum values in time series data 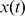.

#### Step 2

Generate the upper and lower envelopes 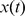 by a cubic spline line and compute the average 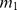. Then, calculate the difference between the time series data 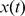 and the mean value 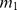. The first difference 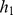
is designed as
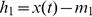
(1)


#### Step 3

Check whether 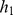 meets the IMF’s conditions. If properties of 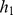 satisfy all the requirements of an IMF, 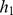 is denoted as the 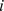 th IMF 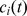 and substitutes the residue 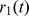 for the original time series data; that is,
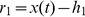
(2)


Otherwise, 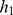 is not an IMF. The above process needs repeating until meeting the filter stop condition and getting the first IMF 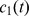, which represents the highest frequency component in the local moment.

#### Step 4

Repeat from *Step 1* to *Step 3.* The sifting process stops when the residue satisfies one of the termination criteria. The original time series data 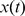 can be described as
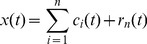
(3)


**Figure 1:**
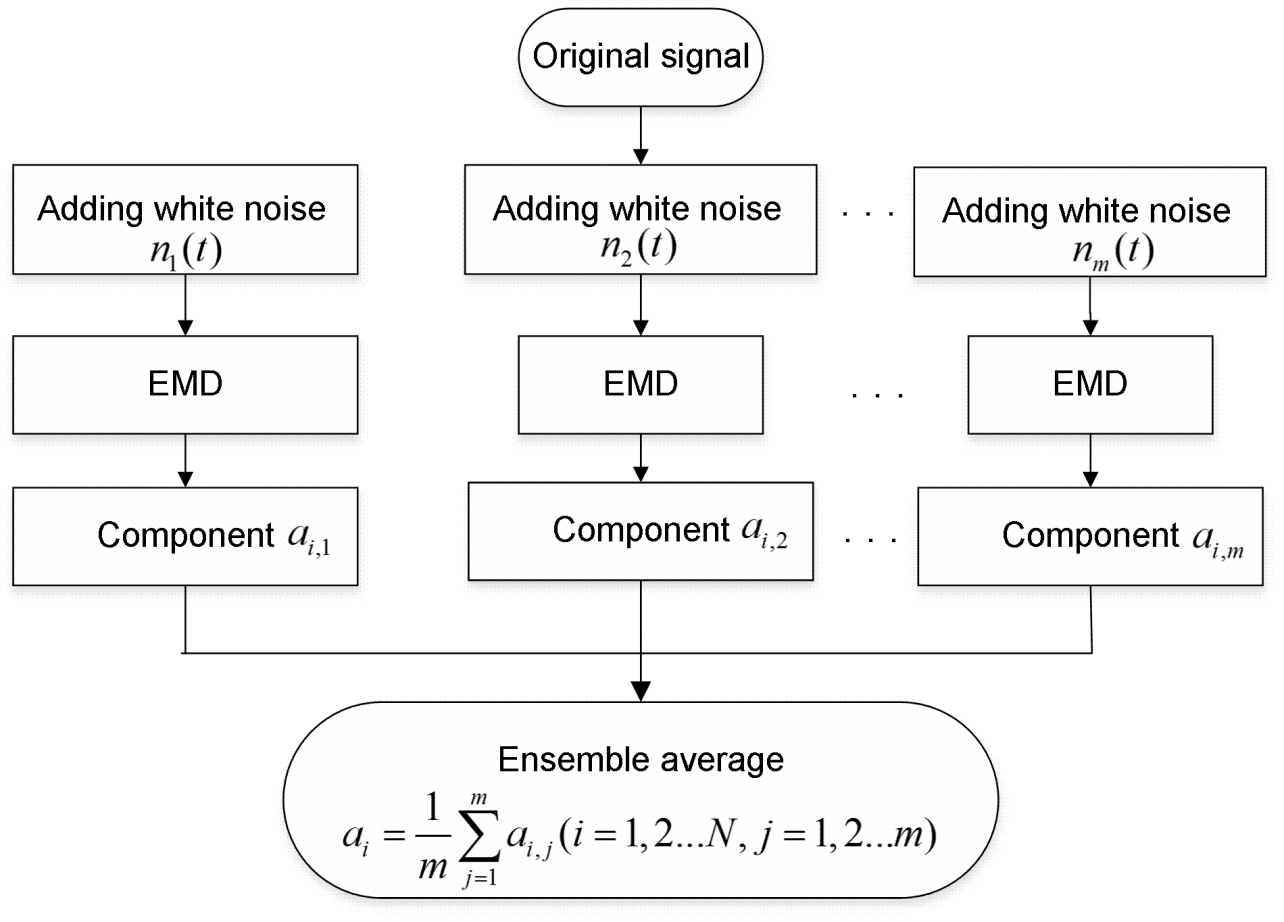
Flowchart of EEMD algorithm.

However, a major shortcoming of the original EMD is the mode mixing, which is defined as a single IMF either consisting of signals with widely disparate scales or a signal of a similar scale residing in different IMF components. To overcome this problem, ensemble empirical mode decomposition (EEMD) was proposed [37], which is a noise-assisted data analysis method. By adding finite white noise signal to the investigated signal, the EEMD method can eliminate the mode mixing problem automatically. The flowchart of EEMD algorithm is shown as Figure 1.

### 2.2 ICA theory

ICA is a potential and promising approach in signal processing. The main concept of this technique lies in unmixing a set of independent sources according to their statistical independency from a linearly mixed input signal. Using the vector-matrix notation, the mathematical model can be represented by the following equation:
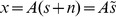
(4)


Where 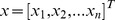 is the observed signal, 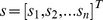 is the source signal, 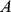 is the 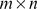 mixing matrix, and 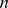 is the noise components same as intrinsic components.

To estimate the sources based on the assumption that they are statistically independent, ICA algorithm considers a linear transformation as following equation:
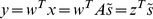
(5)


Where 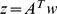, 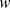 is an estimator of a row of the matrix 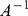, and 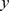 is one of the best estimation of the source signals. Considering the adaptive processing and convergence speed of ICA algorithm, this paper applies the FastICA algorithm [38]–[40] which is based on fixed-point algorithm and is applicable for any type of data to solve the separate matrix. The process of FastICA algorithm is shown as the following steps.

#### Step 1

Center the data 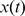 to make its mean zero:
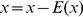
(6)


#### Step 2

Calculate the covariance matrix 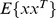.
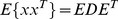
(7)


Where 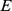 is the orthogonal matrix of eigenvectors of 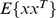 and 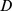 is the diagonal matrix of its eigenvalues, 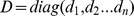
_._

#### Step 3

Whiten the data 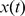 to give the whitening vector 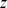:
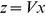
(8)


Where 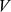 is the whitening matrix, 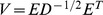
_._

#### Step 4

Choose an initial vector 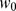 of unit norm.

#### Step 5

Update 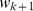:
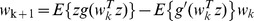
(9)


#### Step 6

Normalize 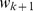:
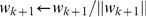
(10)


#### Step 7

If not converged, go back to *Step 5*.

#### Step 8

If converged, calculate one independent component 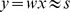.

## Simulation

To validate the effectiveness of the proposed method, simulations are performed. Three source signals are firstly generated as shown in equation (11). Then they are randomly mixed according to equation (12).
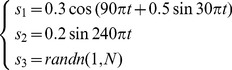
(11)




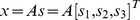
(12)


**Figure 2:**
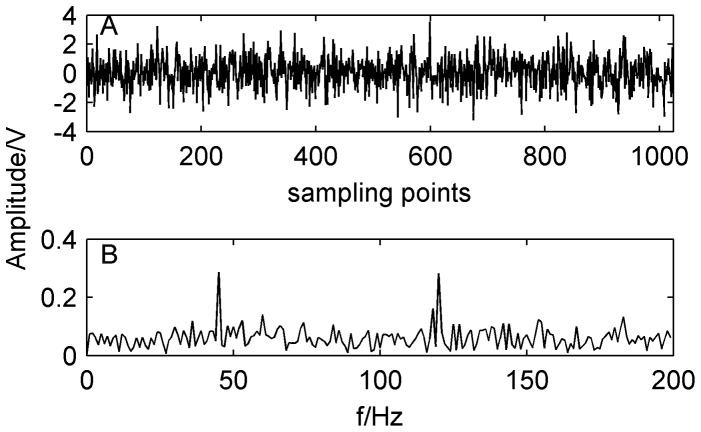
Original simulated signal and its spectrum. A) waveform of original simulated signal; B) spectrum of the original simulated signal.

Figure 2(A–B) shows the mixed signal and its spectrum obtained by the FFT. It can be seen from Figure 2(B) that the fault characteristic frequencies of mixed signals are buried and difficult to be detected in the corresponding spectrum.

**Figure 3:**
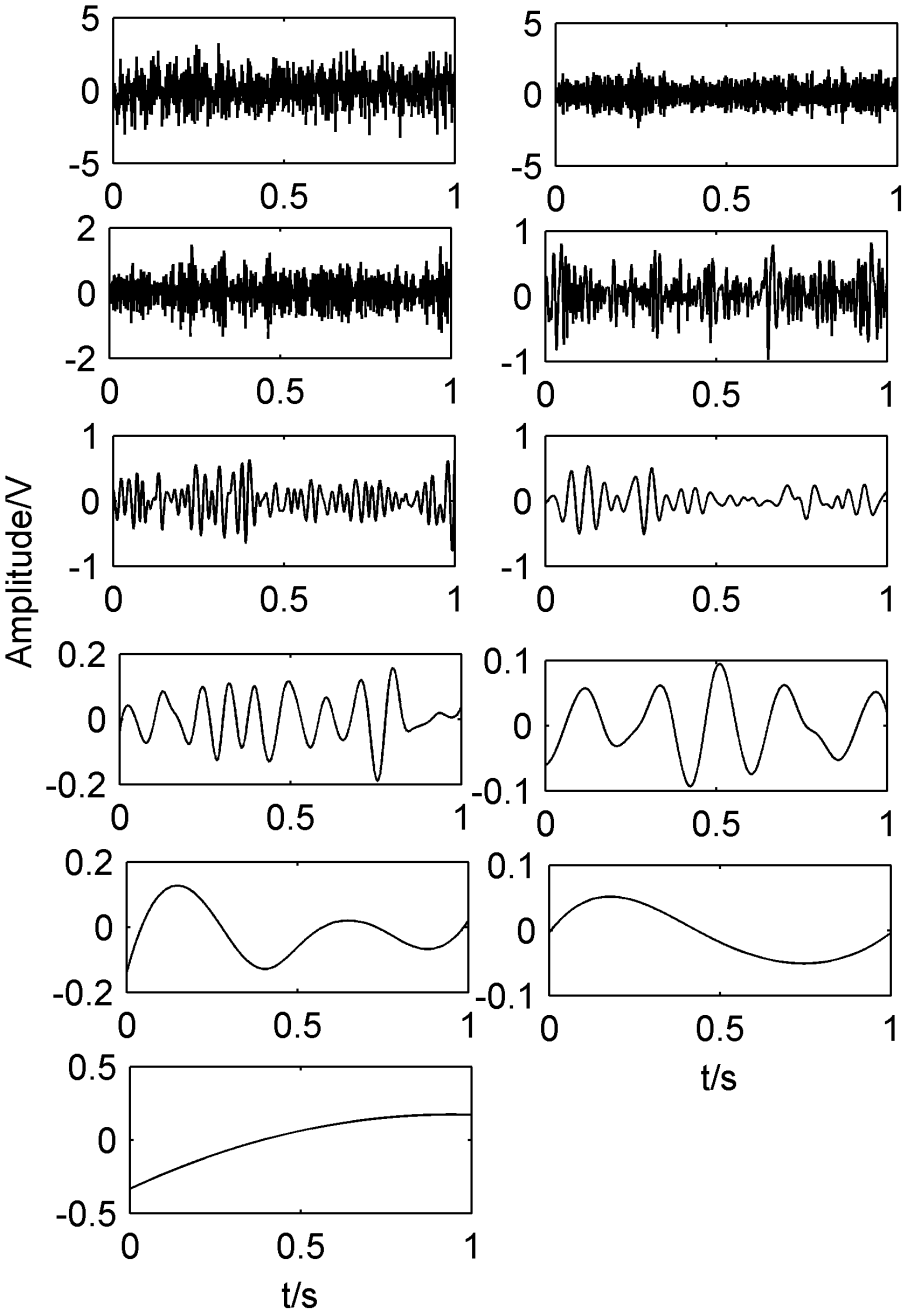
IMF components decomposed by EEMD method.

**Figure 4:**
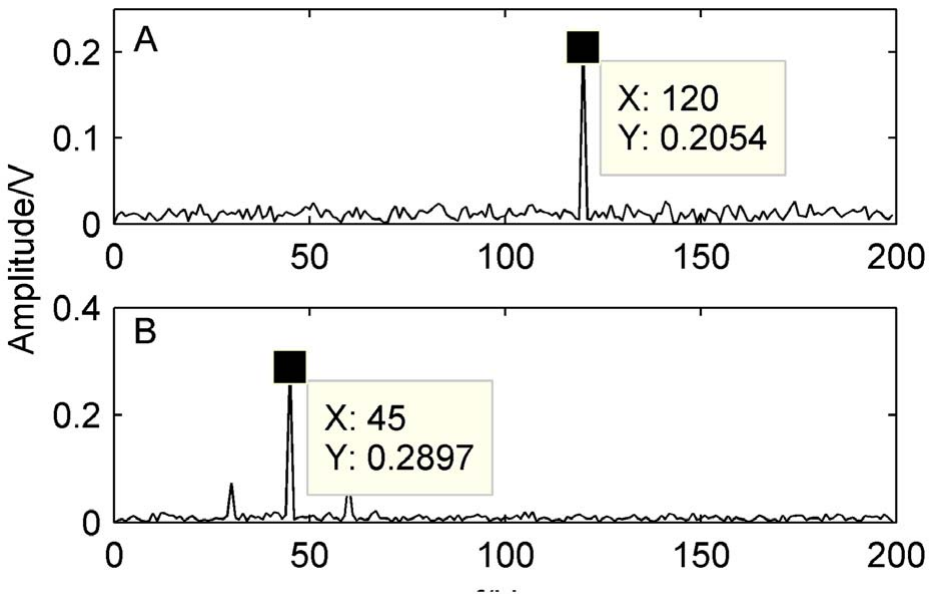
Spectra of separated signals by the proposed method. A) spectrum of IC1; B) spectrum of IC2.

In this section, the above mixed signal is firstly decomposed to IMF by EEMD method shown as Figure 3. Then the cross correlation coefficient of IMF and the original signal is calculated as shown in Table 1. We can see from Table 1 that the cross correlation coefficients of IMF1–IMF5 with the original signal are relatively large and retain more information of the original signal. Therefore, MF1–IMF5 is used as input matrix of FastICA algorithm to separate compound fault. Parts of the separation results are shown in Figure 4(A–B), respectively.

**Table 1:** Cross correlation coefficient of the original simulated signal and IMFs.

**IMF1**	**IMF2**	**IMF3**	**IMF 4**	**IMF 5**	**IMF6**
1	0.3906	0.4880	0.5813	0.5337	0.3943
**IMF7**	**IMF8**	**IMF9**	**IMF10**	**IMF11**	**IMF12**
0.1274	0.0018	0.0017	0.0022	0.0018	0.0017
**IMF 13**	**IMF 14**	**IMF15**	**IMF16**	**IMF17**	**IMF18**
0.0023	0.0008	00096	00096	0.0016	0.0002

From Figure 4, we can see that the mixed signal contains two kinds of fault signals whose fault characteristic frequencies are 120 Hz and 45 Hz. By the proposed method, the compound fault signals are effectively separated from the strong confusion noise signals. Simulation results show that the proposed method can be applied to separate compound fault and extract fault feature of rolling bearings.

## Experiments

Vibration signal analysis usually is one of the most important methods used for condition monitoring and fault diagnosis. However, various factors, such as the complexity of transmission channel, cause that faults that often happen in a rolling bearing are compound faults among the outer race, the inner race, and the rollers.

**Figure 5:**
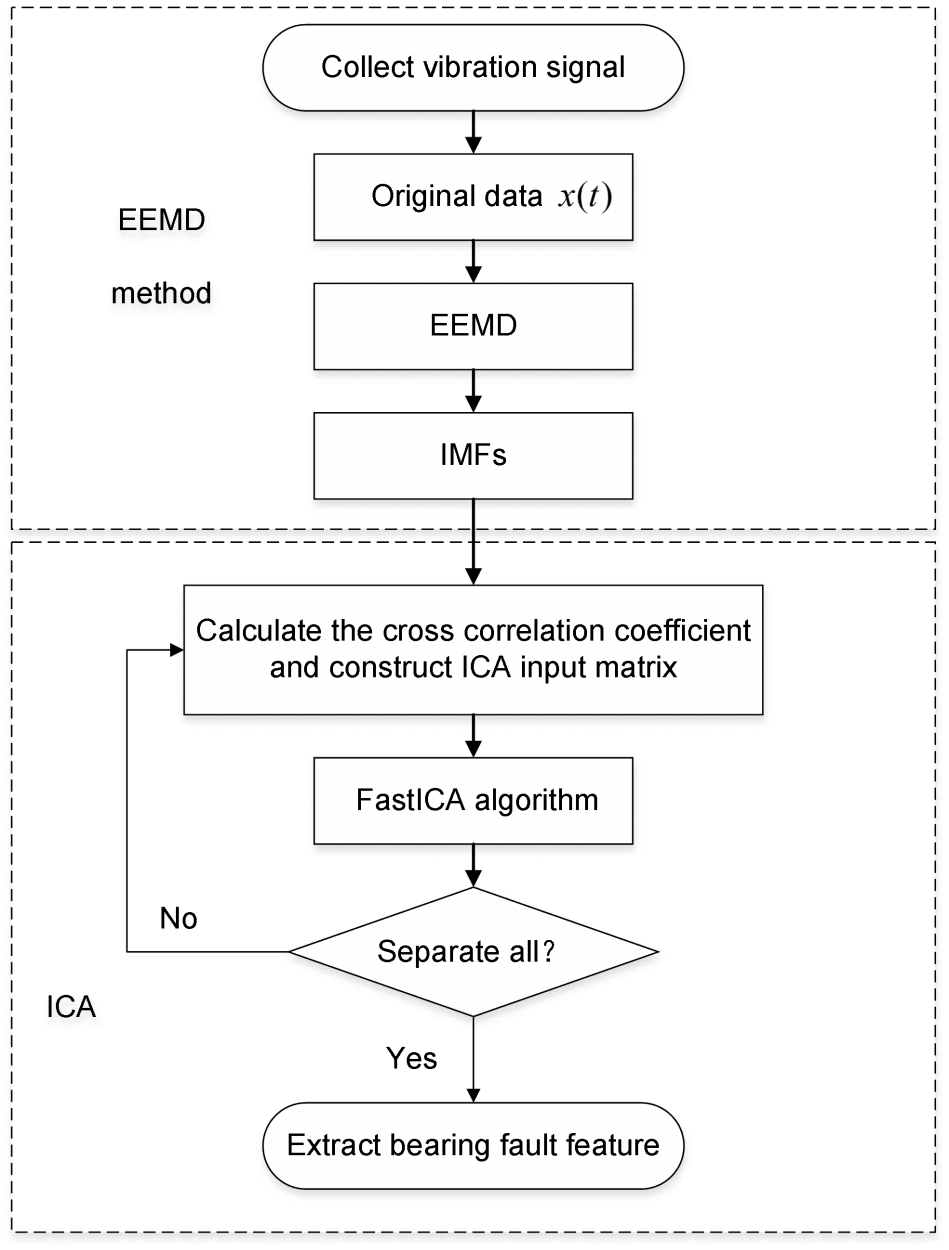
Flowchart of the experiment scheme.

To solve this problem, a novel method of compound fault diagnosis for the rolling bearing based on EEMD method and blind source separation is presented in this paper. In order to verify the efficiency of the proposed methods, several experiments of bearing fault are performed. The detailed experimental scheme is shown in Figure 5. First, taking the compound fault of a bearing outer-race and a roller as the research object, a single channel vibration 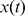 is collected by the acceleration sensor fixed on the bearing seat vertically. Second, decompose the collected vibration signal 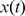 to IMF using EEMD method to obtain multi-channel signals. Third, introduce the cross correlation criterion and select IMF whose cross correlation coefficient is greater with the original signal. Then envelope signal of the chosen IMF is used as the input matrix of ICA to separate bearing compound fault. Lastly, comparing the fault characteristic frequencies in the spectra with theoretical characteristic frequencies of a rolling bearing, the bearing fault features of the equipment are extracted.

In order to evaluate the performance quantitatively, a so-called energy ratio (ER) is defined as following,
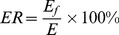
(13)Where 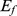 is the energy of the bearing fault signal, 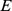 is the total energy. The larger ER value is, the better separation performance will be.

### 4.1 Experiments Setup

**Figure 6:**
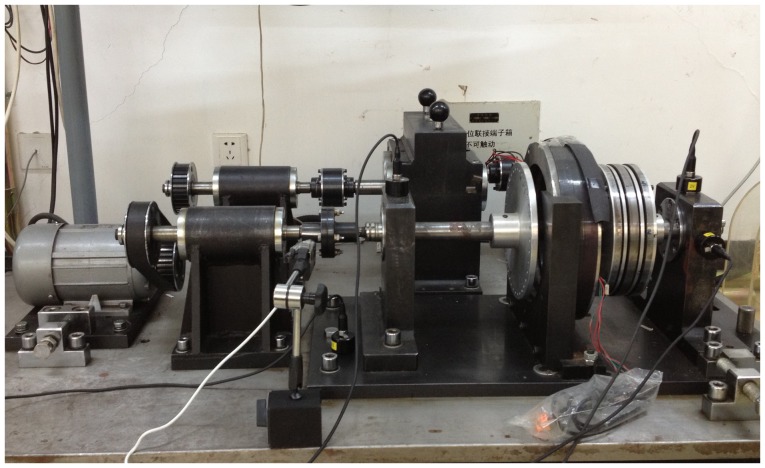
Experimental system for bearing diagnosis.

**Figure 7:**
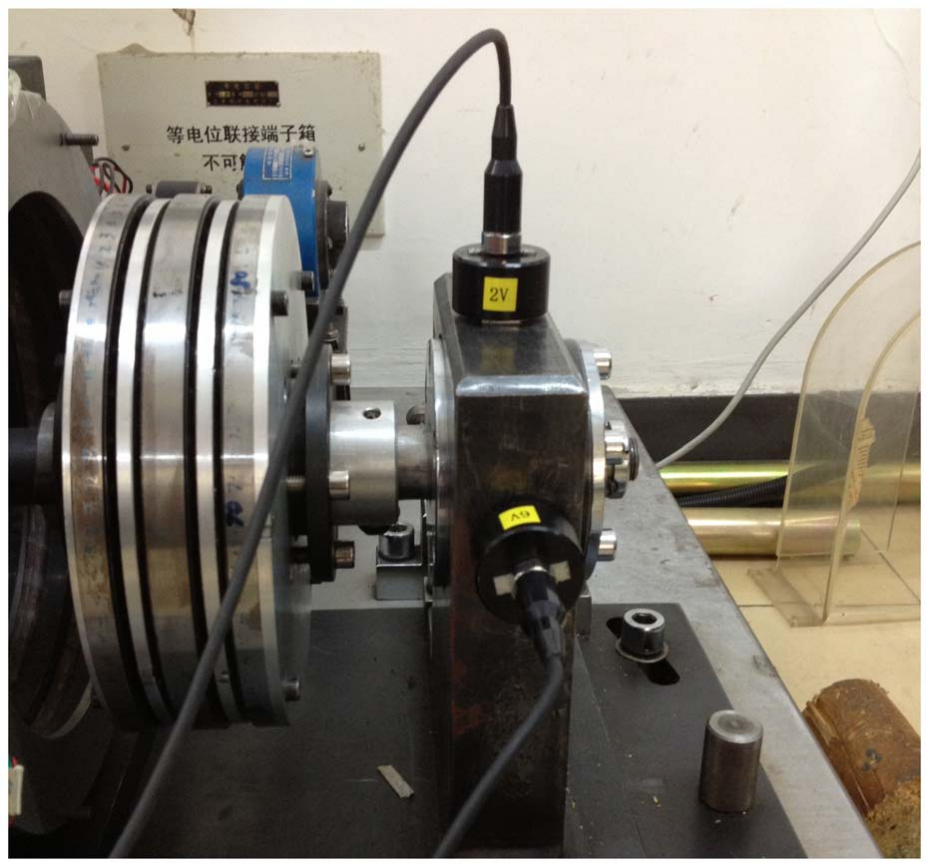
Install location of the acceleration sensor.

Aiming at verifying efficiency of the methods proposed in this paper, the experimental system of bearing fault diagnosis is used. Figure 6 shows the experimental system, including the rotating machine, the rolling bearing and the acceleration sensors. One acceleration sensor is mounted on the bearing housing in the vertical directions to measure the vibration signal of 2V channel, as shown in Figure 7. The faults often occurring in a rolling bearing are compound faults among the outer-race, the inner-race and the rollers. We take the compound fault of bearing outer-race and rollers as the research object,and artificially make those flaws with the use of a wire-cutting machine for the tests of fault diagnosis. The sizes of the flaw are as follows:

Outer-race flaw: 0.5*0.15 mm (width * depth);

Rollers flaw: 0.5*0.15 mm (width * depth).

In order to fully analyze signal features and acquire more comprehensive information for the research of the fault diagnosis, we chose the larger sampling frequency. The sampling frequency is 100 kHz, and the sampling time is 10 s, and the rotating speed of a machine is 500 rpm, 900 rpm and 1300 rpm, respectively.

### 4.2 Characteristic-frequency of a bearing

As mentioned above, faults that often occur in a rolling bearing are usually induced by local defects in the outer race, the inner race, and the rollers. Such defects generate a series of impact vibrations every time a running roller passes over the surfaces of the defects. The characteristic frequencies of a bearing are calculated based on the bearing geometry and the rotor frequency 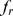. By comparing the fault characteristic frequencies in the spectra with calculated characteristic frequencies of a bearing, the cause of the defect can be identified. For a bearing with a stationary outer race, the characteristic frequencies of a bearing are given by the following equations.

Outer-race defects are revealed at the outer-race pass frequency (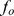):
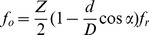
(14)


Rollers defects are revealed at the roller pass-frequency (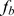):
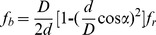
(15)


Where 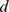 is the diameter of rollers, 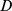 is the pitch diameter, 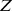 is the number of rollers, 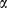 is the contact angle of the rollers, and 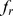 is the rotating frequency. In this work, the calculated characteristic frequencies of the rollers defect and the outer-race defect are shown as Table 2.

**Table 2:** Fault characteristic frequencies of rolling bearing at different speed.

	Fault characteristic frequency		
	500 rpm	900 rpm	1300 rpm
Outer-race	33.2 Hz	59.8 Hz	86.3 Hz
Rollers	39.3 Hz	71.8 Hz	102.3 Hz

### 4.3 Diagnosis by the conventional FFT-based envelope analysis

**Figure 8:**
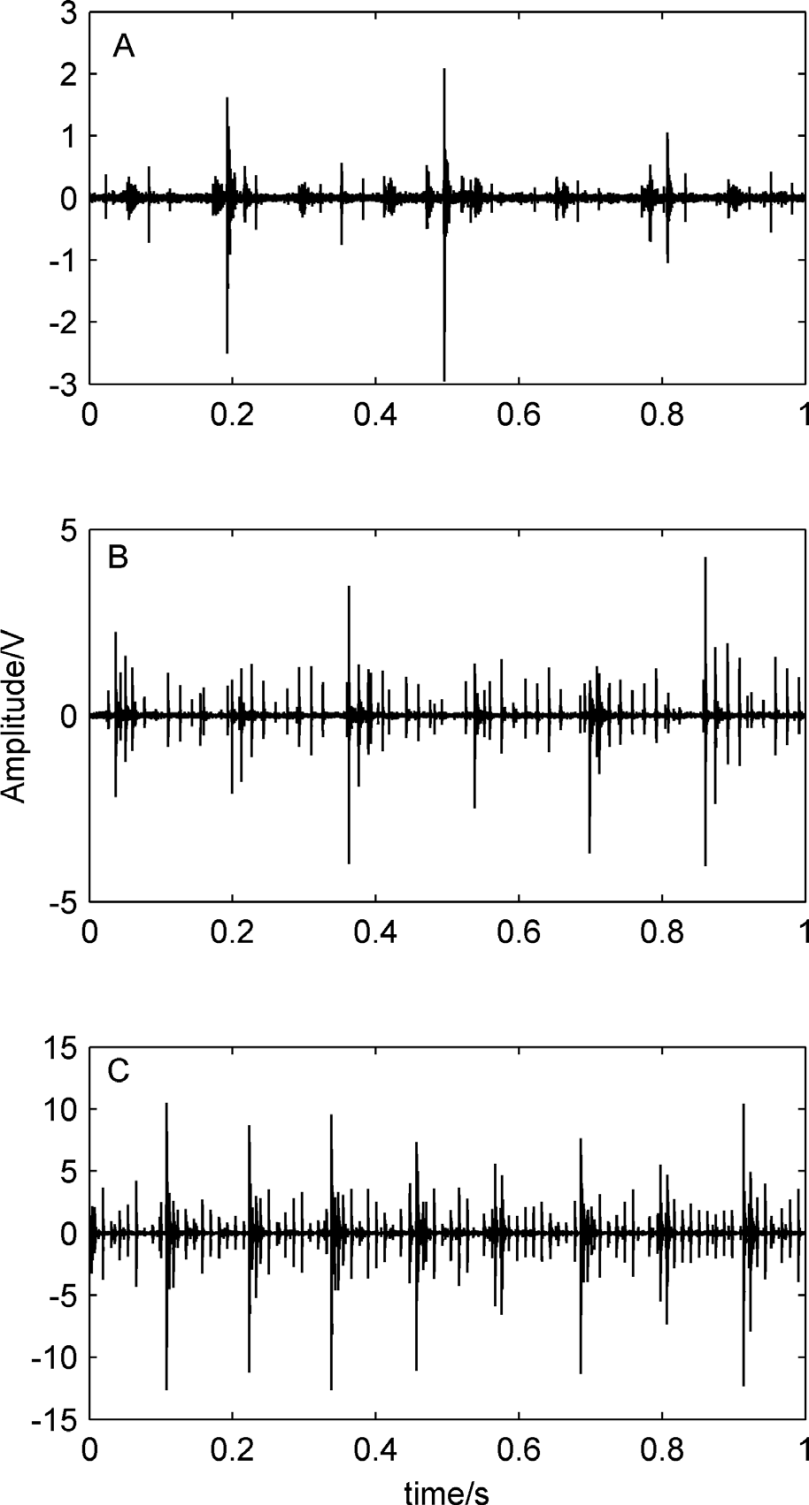
Original diagnosis signal waveforms at different rotating speed. A) at 500 rpm; B) 900 rpm; C) 1300 rpm.

Original diagnosis signals at 500 rpm (see Data S1), 900 rpm (see Data S2) and 1300 rpm (see Data S3) measured by the acceleration sensor mounted on the bearing housing in the vertical directions at different rotating speed are shown in Figure 8(A–C), under the compound fault state of outer-race and rollers. We can see from Figure 8 that there are obvious impulses in the vibration signals. It shows that the experimental system shown as Figure 6 has worked in abnormal state, but the cause and position of the fault is still unknown.

While the bearing failure occurs, the signal of a defect bearing is a typical vibration with amplitude modulation. Therefore, in spectrum analysis, demodulation analysis prior to performing the FFT should be carried out. Envelope detection is usually used for processing the vibration signals with amplitude modulation [41]. To implement the envelope detection technique, the Hilbert transform is often applied in vibration signal demodulation.

**Figure 9:**
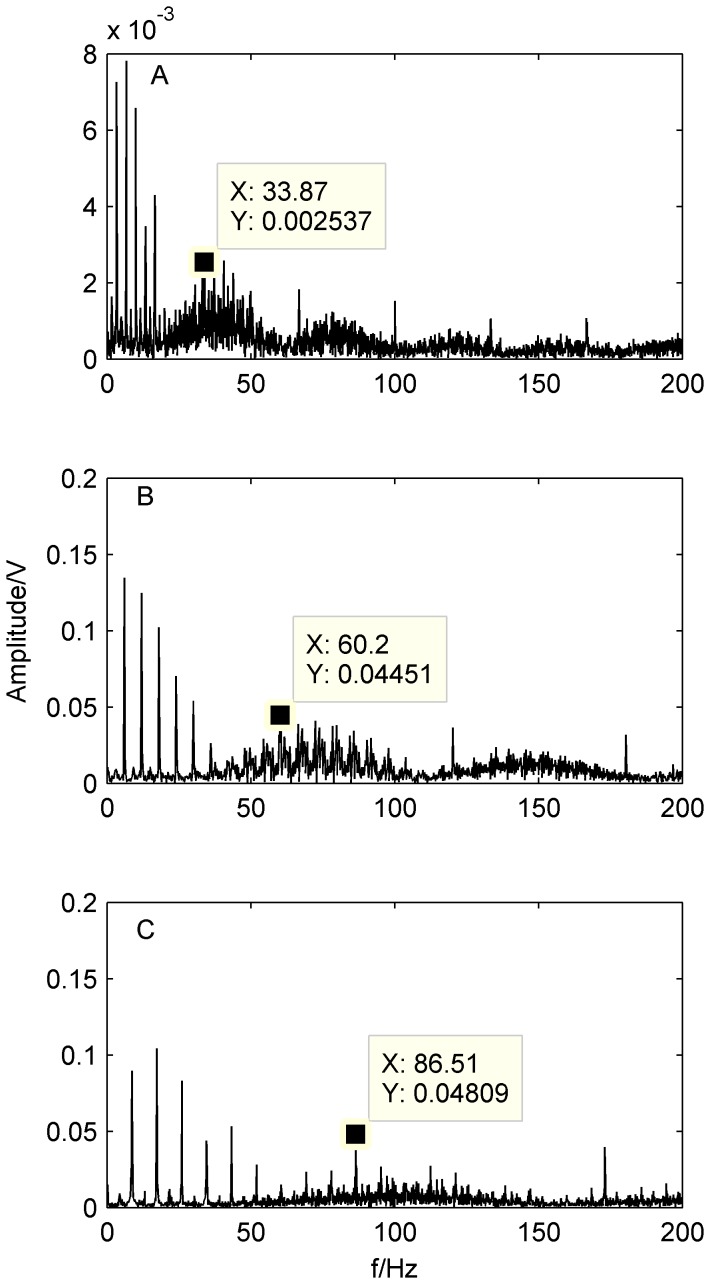
Envelope spectra of the original signal at different rotating speed. A) 500 rpm; B)900 rpm; B)1300 rpm.

In the present work, the FFT-based Hilbert transform is considered. Figure 9(A–C) shows the envelope spectra of bearing faults obtained by the FFT with Hilbert-transform based envelope.

As shown in Figure 9(B), characteristic frequency 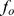 at 60.2 Hz is not obviously apparent, but the outer-race defect of a bearing could still be identified by the calculated characteristic frequency (59.8 Hz). We also can see from Figure 9(B) that 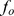 is a little larger than the characteristic frequency. The same conclusion could be obtained from Figure 9(A) and Figure 9(C). The reasons are explained as following. (1) Equations (11–12) are based on the assumption of a pure rolling motion. However, in practice, some sliding motion may occur, which causes slight deviation of the characteristic frequency locations; (2) The rotating speed fluctuates around the set value frequency Therefore, the calculated equations should be regarded as approximations only.

Because the machine contains a strong noise component and various faults occur simultaneously, the fault characteristic frequency of the rollers defect is buried and difficult to identify in the spectrum, where all the types of bearing characteristic frequencies should be located. Therefore, bearing compound faults cannot be completely detected by the conventional envelope analysis technique.

### 4.4 Diagnosis by the wavelet analysis

Wavelet transform [42] is a relatively effective analysis method in fault diagnosis of rotating machinery and has received considerable attention for its potential applications during the past decade. The method is actually to decompose original signal 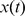 into sub-signals with different frequency bands through basis function 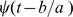, in which 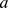 is a scale factor that controls contraction and stretch of the waveform, and 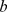 stands for the time-shift factor. The wavelet transform of signal 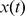 can be defined as
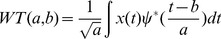
(16)


From the equation (13), we can see that the wavelet transform of a signal is essentially equivalent to observe the signal through the changes of wavelet scale factor and time-shift factor. Because the data that computers store and process is in binary format, discrete wavelet transform (DWT) is quite suitable for the rolling bearing fault diagnosis. Therefore, the DWT is considered and applied to the bearing compound fault diagnosis in this study.

**Figure 10:**
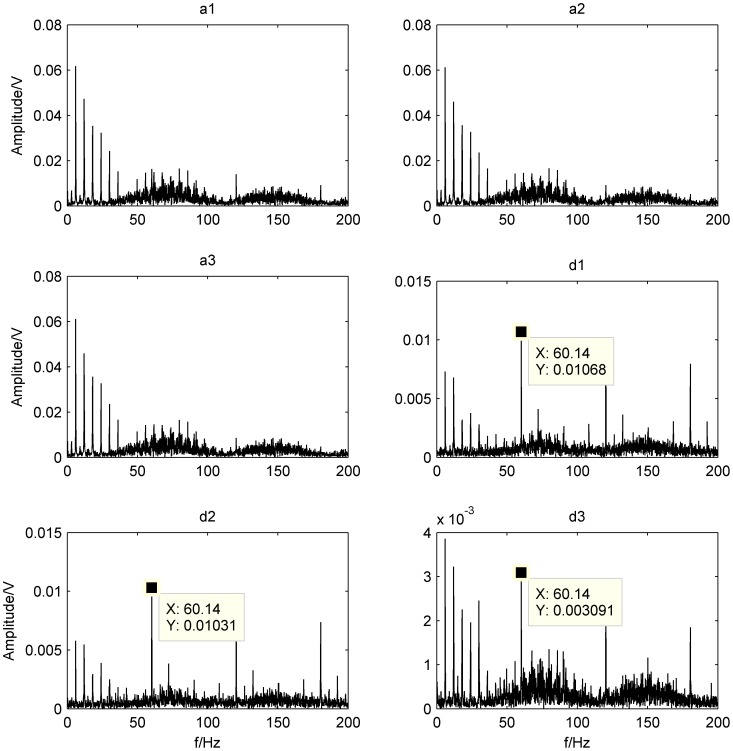
Envelope spectra of each level wavelet coefficients.

In the present work, compound fault of outer-race and rollers at 900 rpm is analyzed. Wavelet basis function is dB4 and decomposition level is 3. After performing the DWT, high-frequency wavelet coefficients 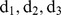 and low-frequency wavelet coefficients 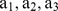 are obtained, respectively. The corresponding envelope spectra of each level wavelet coefficients achieved by the FFT with Hilbert-transform are shown in Figure 10.

It can be seen from Figure 10 that the frequency 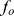 is about 60.14 Hz, similar to the calculated characteristic frequency of the outer-race defect at 59.8 Hz; hence, it can be judged as the outer-race defect. Although more confusion noises are still observed in these spectra, the feature of the outer-race defect can be obviously detected comparing with the envelope spectrum of the original signal shown in Figure 9. The features of other faults (rollers defect and unbalance fault) are not as easy to extract as the outer-race in the Figure 10. Therefore, in this case, the compound faults are difficult to separate by DWT method.

### 4.5 Diagnosis by the proposed method

In this section, the single channel vibration signal collected by acceleration sensor at 900 rpm is decomposed to IMF by EEMD method to obtain multi-channel signals for under-determined blind source separation, i.e., observed signal numbers are less than sources numbers. After implementing the EEMD method, multiple IMFs are acquired. Because EEMD will generate excessive decomposition and false components in the course of calculation, the cross correlation coefficient of IMF and the original signal is calculated and shown in Table 3.

**Table 3:** Cross correlation coefficient of the simulated signal and IMFs.

**IMF1**	**IMF2**	**IMF3**	**IMF 4**	**IMF 5**	**IMF6**
1	0.7395	0.5044	0.3803	0.3209	0.2205
**IMF7**	**IMF8**	**IMF9**	**IMF10**	**IMF11**	
0.1132	0.0591	0.0322	−0.0565	0.0867	

**Figure 11:**
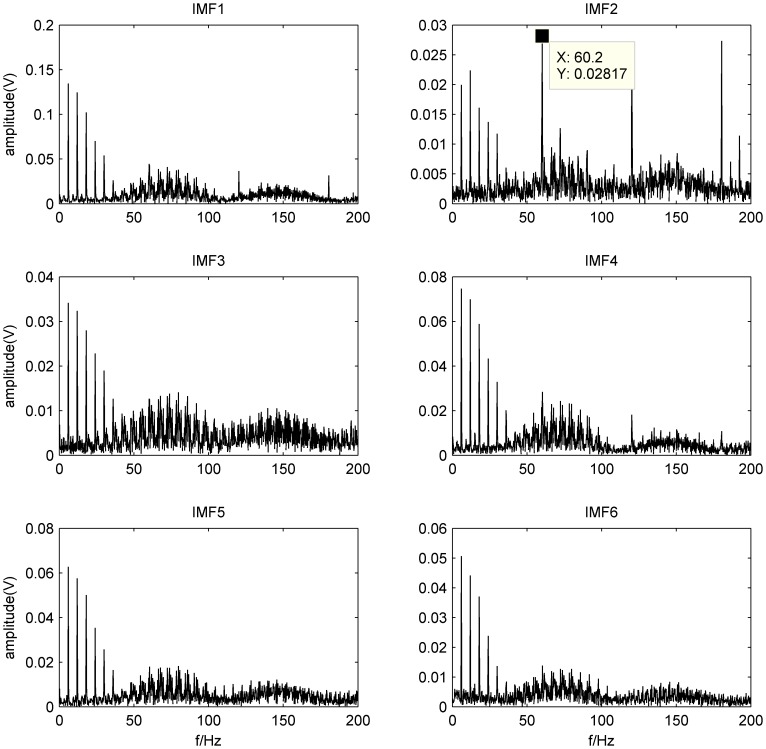
Envelop spectra of IMF1–IMF6.

It can be estimated from Table 3 that there exist large quantity of false components whose cross correlation coefficient with the original signal is extremely small and retain less information of the original signal. Therefore, IMF1–IMF6 is selected to solve the problem of under-determined blind source separation in this study. Figure 11 shows the envelop spectra of IMF1–IMF6, respectively.

Similarly, we can see from Figure 11 that the outer-race defect is easily identified, but the features of other faults cannot be observed. It is indicated that the wavelet analysis and EEMD method is more effective in outer-race defect diagnosis than the FFT technique, because the two methods, as a effective time-frequency analysis method for observing the time-frequency property of sub-band signals, make the local feature more obvious and clear. However, the bearing compound faults cannot still be separated by these methods.

**Figure 12:**
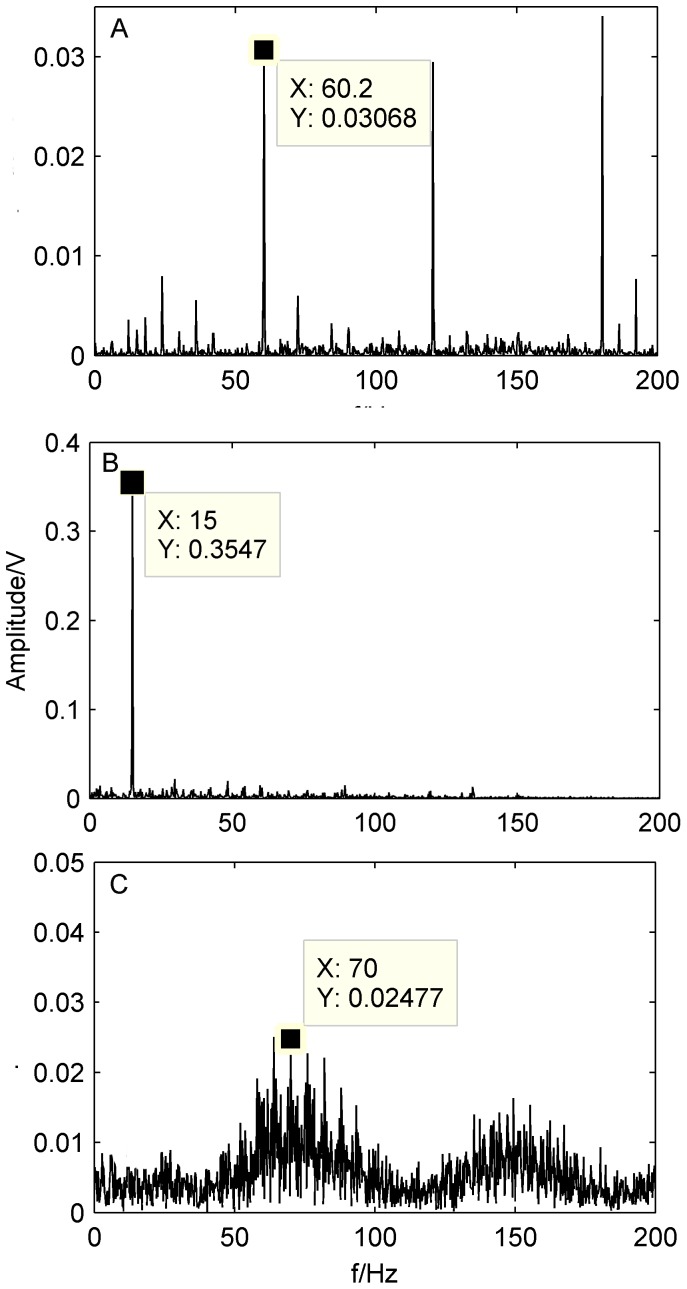
Spectra of the separated signals by the proposed method at 900 rpm. A) spectrum of the outer-race defect; B) spectrum of the unbalance fault; C) spectrum of the rollers defect.

For the purpose of identifying the fault signal which consists of various fault features, it is essential to utilize ICA technique to separate source signals from the observed signal. The specific diagnostic procedure is given as follows. First, we obtain the envelop signals from IMF1–IMF6 through Hilbert-transform, respectively, because the IMF obtained by EEMD method is still amplitude modulation signal. Second,the obtained envelop signals form a 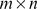 matrix, where 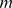 represents the number of input signals and 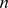 represents the number sampling points. Third, the matrix is used as the input matrix of ICA to separate fault signal by FastICA algorithm. Finally, we diagnosed the conditions of bearings by the extracted fault features. Parts of the verification results are shown in Figure 12(A–C), respectively.

Figure 12(A) shows the spectrum of the bearing outer-race defect. It can be seen from Figure 12A that the frequency 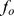 at 60.2 Hz can be obviously observed and is very close to the calculated characteristic frequency of the outer-race defect at 59.8 Hz. Therefore, it can be easily identified as the outer-race defect by the spectrum.

Figure 12(B) shows the spectrum of the system unbalance fault. The frequency 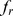 is clearly shown in Figure 12(B). The frequency 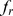 is about 15 Hz, equal to the rotating frequency at 15 Hz; hence, it can be judged as the system unbalance fault.

Figure 12(C) shows the spectrum of the bearing rollers defect. The characteristic frequency 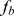 at 72.4 Hz appears in the spectrum, as shown in Figure 12(C). The frequency 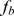 is similar to the calculated characteristic frequency of the bearing rollers defect at 71.8 Hz. It also shows that more confusion noises still exist in the spectrum, which makes the rollers defect more difficultly diagnose than the outer-race defect and unbalance fault.

**Figure 13:**
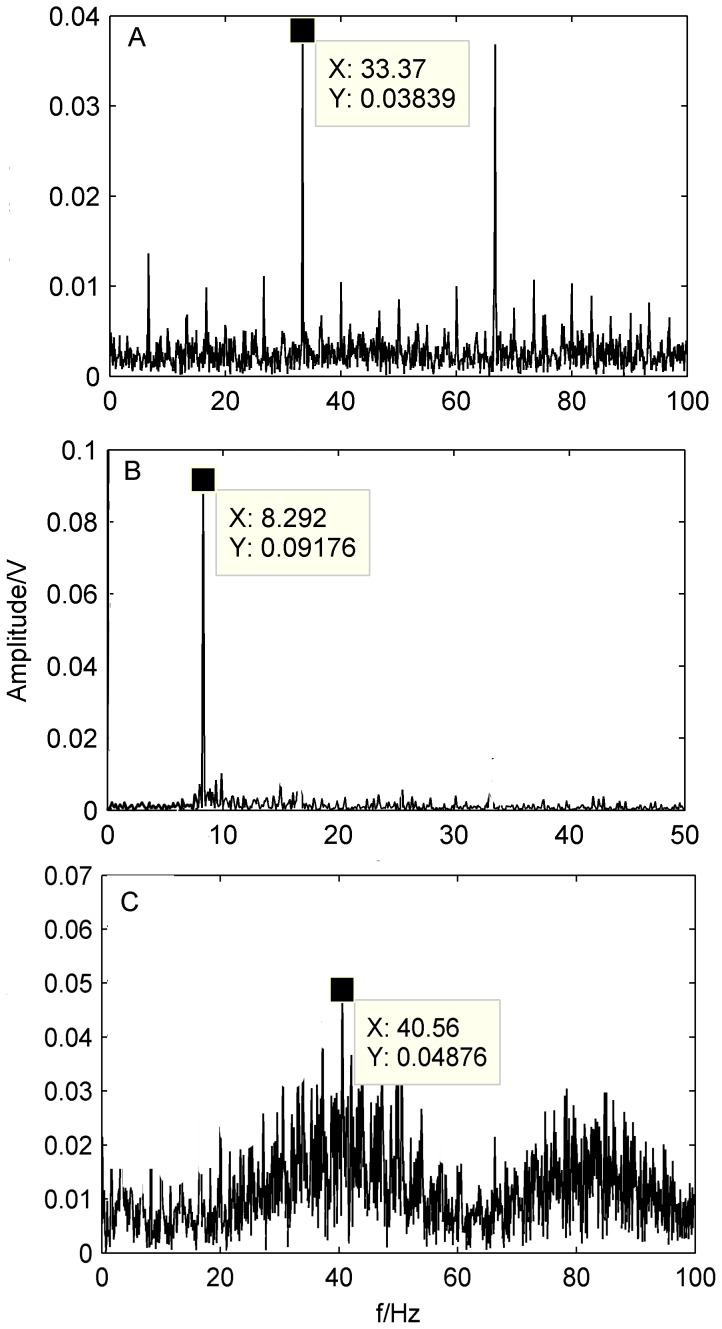
Spectra of the separated signals by the proposed method at 500 rpm. A) Spectrum of the outer-race defect; B) Spectrum of the unbalance fault; C) Spectrum of the rollers defect.

**Figure 14:**
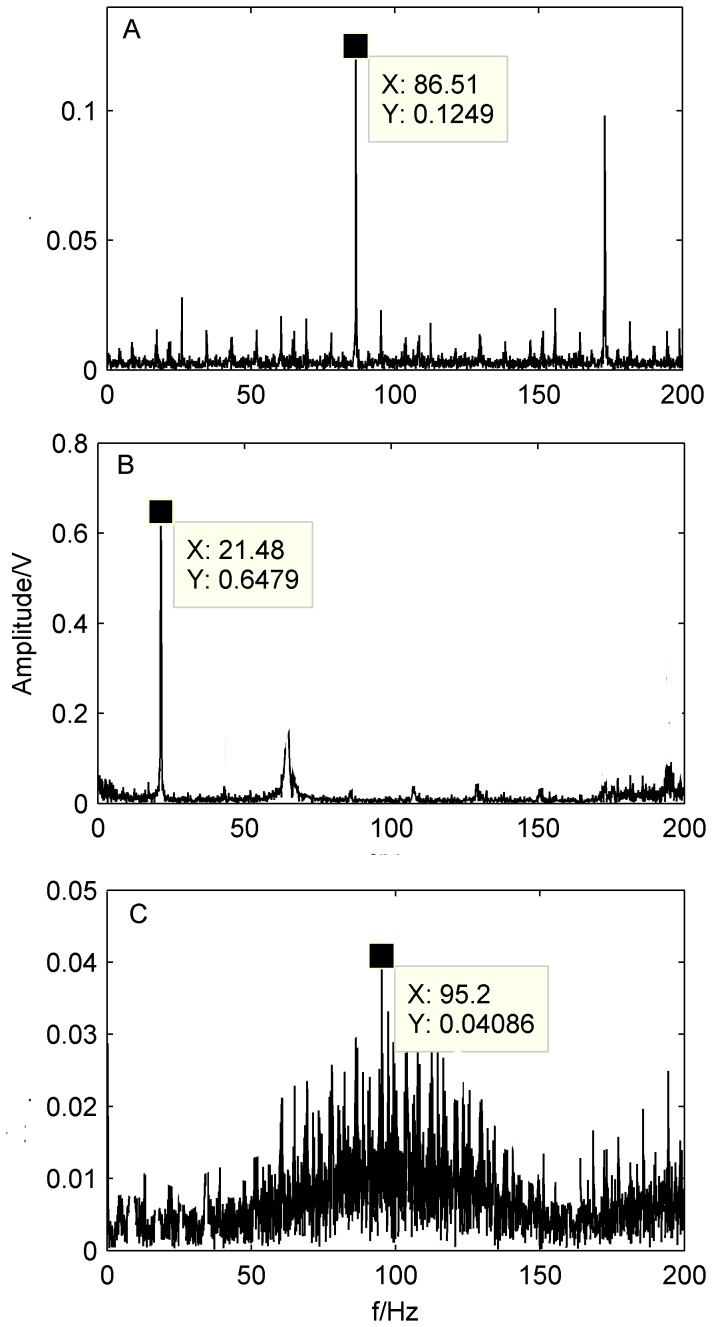
Spectra of the separated signals by the proposed method at 1300 rpm. A) Spectrum of the outer-race defect; B) Spectrum of the unbalance fault; C) Spectrum of the rollers defect.

Spectra of the bearing outer-race defect, rollers defect and the system unbalance fault at 500 rpm and 1300 rpm are shown as Figure 13(A–C) and Figure 14(A–C), respectively. It also can be seen from Figure 13–14 that the out-race defect and the unbalance fault can be obviously identified by the proposed method, whereas, it is not much effective for the rollers defects.

In order to further verify the validity of the proposed method, we select energy ratio as a statistical measure to compare conventional methods with the proposed method in a quantitative way. Table 4 shows the calculated results. We can see from Table 4 that energy ratio calculated by the proposed method is significantly larger than conventional methods. It illustrates that fault characteristic frequency is more obvious and compound faults have been extracted.

**Table 4:** Energy ratio calculated by the different methods.

		Energy ratio			
		FFT	Wavelet analysis	EEMD	EEMD-ICA
500 rpm	Outer-race	1.89	25.39	30.25	73.36
	Rollers	1.20	7.13	9.56	21.36
	Unbalance	36.32	56.78	60.23	85.12
900 rpm	Outer-race	2.29	21.86	35.86	80.70
	Rollers	0.72	0.63	2.63	25.68
	Unbalance	23.33	45.63	62.73	89.47
1300 rpm	Outer-race	4.26	19.63	31.89	83.26
	Rollers	1.23	2.10	4.33	19.23
	Unbalance	27.45	62.30	45.32	80.25

According to the results, we can conclude that the vibration signal collected by sensor in the experimental system shown as Figure 6 contains outer-race defect, rollers defect and unbalance fault, respectively. As mentioned in the previous section, the proposed method is not much effective for the rollers defects. It can be explained as follows. First, the bearing outer-race is stationary, but the rollers are rotary when the experimental equipment in the operating condition, which makes the features of the signal in the rollers defect measured by the acceleration sensor more difficult to extract than in the outer-race defect. Second, the rollers revolve around the rotary shaft along with their rotation, respectively, so the signal collected in the rollers defect is more complicated comparing with in the outer-race defect. For those conditions, the problem will be further discussed and solved in the future work.

## Conclusion

In this paper, a new method combined EEMD with ICA technique is proposed to effectively extract compound fault features of the rolling bearing. Comparing with the conventional FFT-based Hilbert transform, the wavelet analysis, and EEMD method, the results have shown that the compound faults, such as the bearing outer-race defect, the roller defect, and the unbalance fault of experimental system have been effectively separated by the proposed method. In future work, optimization of the proposed method will be focus on for further improving.

## Supporting Information

Data S1 Original diagnosis signal at 500 rpm.
(ZIP)

Data S2 Original diagnosis signal at 900 rpm.
(ZIP)

Data S3 Original diagnosis signal at 1300 rpm.
(ZIP)
